# Supplementary material for: UBR4/POE facilitates secretory trafficking to maintain circadian clock synchrony
Source: Nat Commun. 2022 Mar 24;13:1594. doi: 10.1038/s41467-022-29244-1 (PMC8948264; doi:10.1038/s41467-022-29244-1)
Supplement: Supplementary file 1 — Supplementary Information [file 41467_2022_29244_MOESM1_ESM.pdf]

SUPPLEMENTARY INFORMATION

UBR4/POE facilitates secretory trafficking to maintain circadian clock synchrony

Sara Hegazi,[1,2] Arthur H. Cheng,[1,2] Joshua J. Krupp,[1] Takafumi Tasaki,[3,4] Jiashu Liu,[1,2] Daniel A. Szulc,[5,6] Harrod H. Ling,[1,2#]Julian Rios Garcia,[1,2] Shavanie Seecharran,[1,2] Tayebeh Basiri,[7] Mehdi Amiri,[7] Zobia Anwar,[1,2] Safa Ahmad,[1] Kamar Nayal,[1,2] Nahum Sonenberg,[7] Bao-hua Liu,[1,2] Hai-Ling Margaret Cheng,[5,6,8] Joel D. Levine,[1,2,9*] Hai-Ying Mary Cheng[1,2*]

[1] Department of Biology, University of Toronto Mississauga, Mississauga, ON L5L 1C6, Canada

[2] Department of Cell and Systems Biology, University of Toronto, Toronto, ON M5S 3G5, Canada

[3] Division of Protein Regulation Research, Medical Research Institute, Kanazawa Medical University, Uchinada, Ishikawa 920-0293, Japan

[4] Department of Medical Zoology, Kanazawa Medical University, Uchinada, Ishikawa 920-0293, Japan

[5] Institute of Biomedical Engineering, University of Toronto, Toronto, ON M5S 3G9, Canada

[6] Ted Rogers Centre for Heart Research, Translational Biology & Engineering Program, University of Toronto, Toronto, ON M5G 1M1, Canada

[7] Department of Biochemistry, Goodman Cancer Research Center, McGill University, Montreal, QC H3A 1A3, Canada

[8] The Edward S. Rogers Sr. Department of Electrical & Computer Engineering, University of Toronto, Toronto, ON M5S 3G4, Canada

[9] Department of Ecology and Evolutionary Biology, University of Toronto, Toronto, ON M5S 3B2

*Correspondence should be directed to haiying.cheng@utoronto.ca and joel.levine@utoronto.ca

[#]deceased

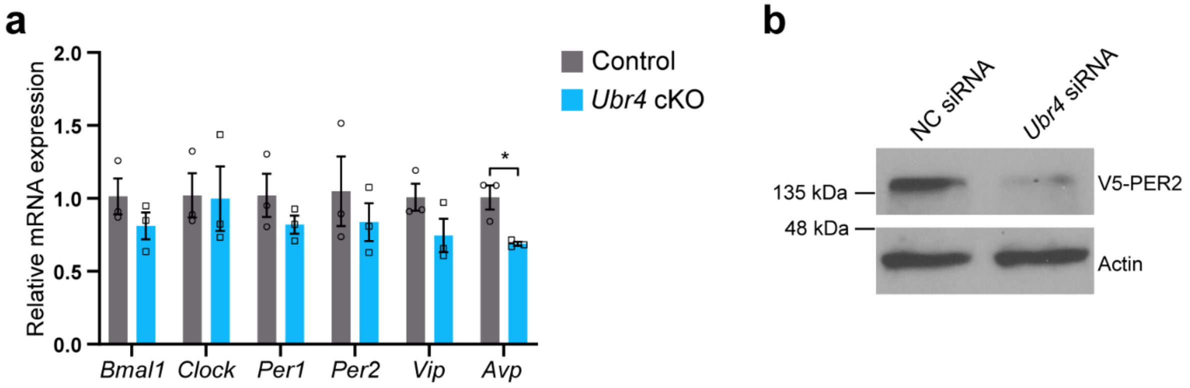

Supplementary Figure 1. Effects of *Ubr4* ablation or knockdown on clock gene expression. **a** qRT-PCR analyses of clock gene expression in the SCN at CT 9. Values represent mean ± SEM. n = 3 samples per group. Two-tailed unpaired t-test conducted for each gene. *p = 0.0189 vs. control. **b** Western blot of lysates from Neuro-2a cells that had been transfected with V5-PER2 along with either negative control (NC) siRNA or *Ubr4* siRNA. Antibodies against V5 (top) and actin (bottom) were used. Source data are provided as a Source Data file.

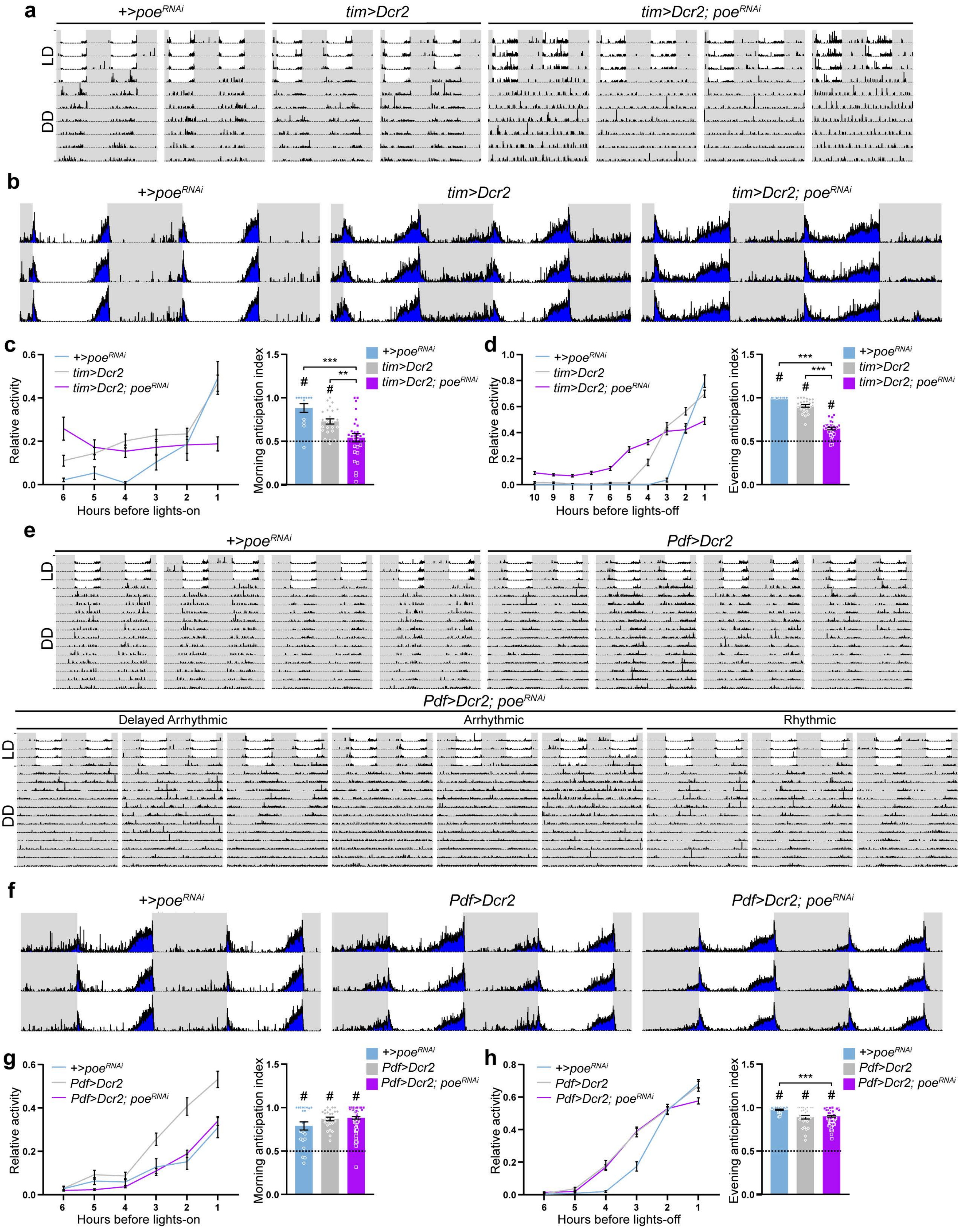

a
+>poe^RNAi
tim>Dcr2
tim>Dcr2; poe^RNAi
LD
DD
b
+>poe^RNAi
tim>Dcr2
tim>Dcr2; poe^RNAi
c
Relative activity
Hours before lights-on
Morning anticipation index
+>poe^RNAi
tim>Dcr2
tim>Dcr2; poe^RNAi
***
**
#
d
Relative activity
Hours before lights-off
Evening anticipation index
+>poe^RNAi
tim>Dcr2
tim>Dcr2; poe^RNAi
***
***
#
e
+>poe^RNAi
Pdf>Dcr2
LD
DD
Pdf>Dcr2; poe^RNAi
Delayed Arrhythmic
Arrhythmic
Rhythmic
f
+>poe^RNAi
Pdf>Dcr2
Pdf>Dcr2; poe^RNAi
g
Relative activity
Hours before lights-on
Morning anticipation index
+>poe^RNAi
Pdf>Dcr2
Pdf>Dcr2; poe^RNAi
#
h
Relative activity
Hours before lights-off
Evening anticipation index
+>poe^RNAi
Pdf>Dcr2
Pdf>Dcr2; poe^RNAi
***
#

Supplementary Figure 2. *Poe* knockdown in *D. melanogaster* clock neurons impairs behavioral rhythms. a, e Representative actograms of individual *tim>Dcr2; poe*^{RNAi} flies and controls (**a**), or individual *Pdf>Dcr2; poe*^{RNAi} flies and controls (**e**), under 12:12 LD and DD conditions. **b, f** Averaged actograms of *tim>Dcr2; poe*^{RNAi} flies and controls (**b**), or *Pdf>Dcr2; poe*^{RNAi} flies and controls (**f**), under 12:12 LD conditions. **c** Mean relative activity counts during the hours preceding lights-on (left), and quantification of morning anticipation index (right) of *tim>Dcr2; poe*^{RNAi} flies and controls under LD conditions. +>*poe*^{RNAi}: n = 13, *tim>Dcr2:* n = 25, *tim>Dcr2; poe*^{RNAi}: n = 28. $^{\#}p<0.0001$ by one-sample t-test compared to the value of 0.5; values greater than 0.5 indicate anticipatory behavior. $^{**}p = 0.0041$, $^{***}p<0.0001$ vs. controls; one-way ANOVA with Bonferroni's post hoc. **d** Mean relative activity counts during the hours preceding lights-off (left), and quantification of evening anticipation index (right) of *tim>Dcr2; poe*^{RNAi} flies and controls under LD conditions. +>*poe*^{RNAi}: n = 13, *tim>Dcr2*: n = 26, *tim>Dcr2; poe*^{RNAi}: n = 28. $^{\#}p<0.0001$ by one-sample t-test compared to the value of 0.5; values greater than 0.5 indicate anticipatory behavior. $^{***}p<0.0001$ as indicated; Kruskal-Wallis with a Dunn's post hoc. (Note: SEM of the mean evening anticipation index of +>*poe*^{RNAi} flies = 0.0003). **g** Mean relative activity counts during the hours preceding lights-on (left), and quantification of morning anticipation index (right) of *Pdf>Dcr2; poe*^{RNAi} flies and controls under LD conditions. +>*poe*^{RNAi}: n = 22, *Pdf>Dcr2:* n = 27, *Pdf>Dcr2; poe*^{RNAi}: n = 88. $^{\#}p<0.0001$ by one-sample t-test compared to the value of 0.5; values greater than 0.5 indicate anticipatory behavior. Kruskal-Wallis with a Dunn's post hoc was used for between group testing. **h** Mean relative activity counts during the hours preceding lights-off (left), and quantification of evening anticipation index (right) of *Pdf>Dcr2; poe*^{RNAi} flies and controls under LD conditions. +>*poe*^{RNAi}: n = 26, *Pdf>Dcr2:* n = 28, *Pdf>Dcr2; poe*^{RNAi}: n = 90. $^{\#}p<0.0001$ by one-sample t-test compared to the value of 0.5; values greater than 0.5 indicate anticipatory behavior. $^{***}p<0.0001$ as indicated; Kruskal-Wallis with a Dunn's post hoc. Values represent mean ± SEM. 'n' represents number of flies. For all actograms, white and gray regions represent the light and dark periods, respectively. Source data are provided as a Source Data file.

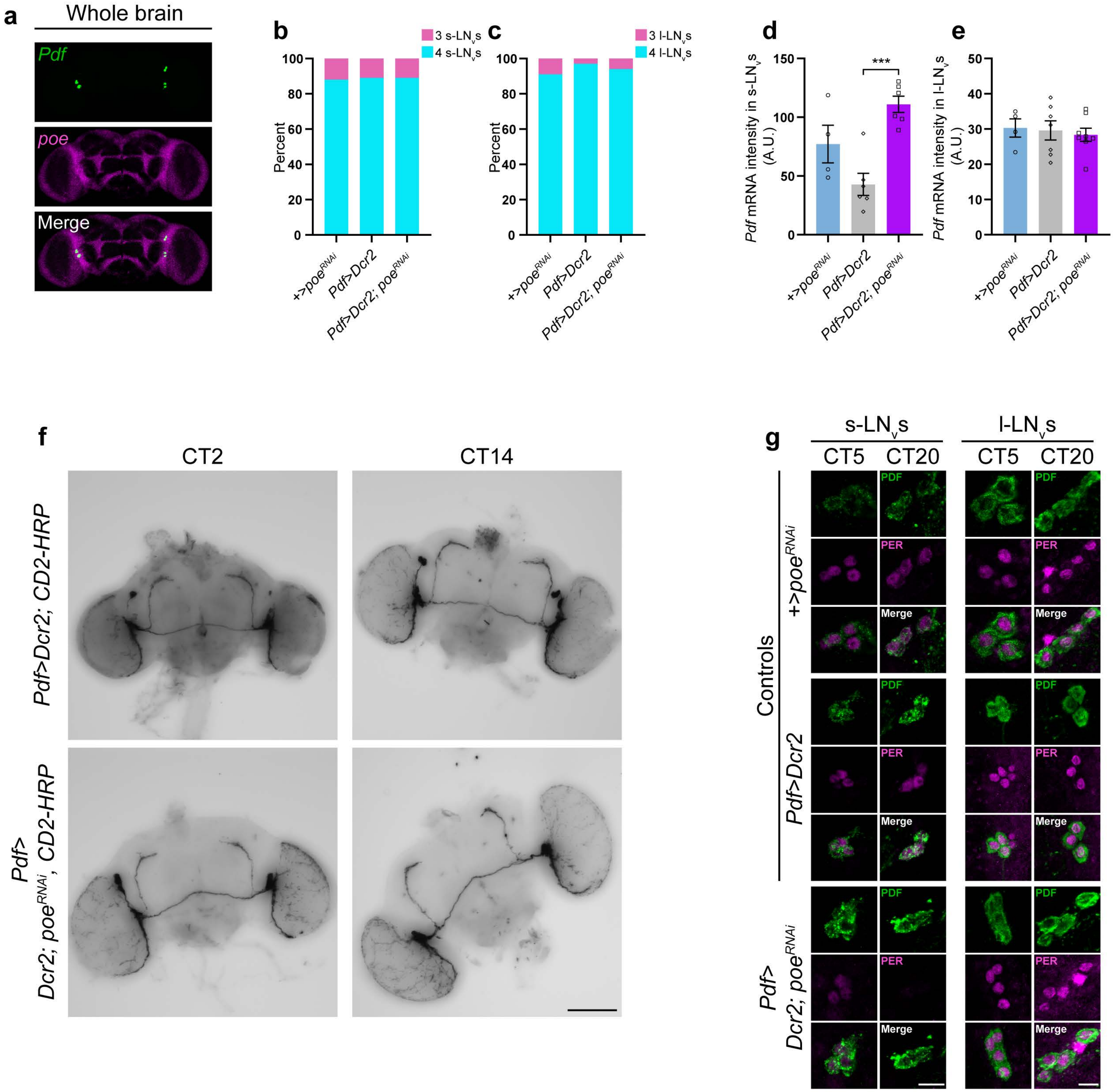
a
Whole brain
Pdf
poe
Merge
b
3 s-LNvs
4 s-LNvs
Percent
+>poeRNAi
Pdf>Dcr2
Pdf>Dcr2; poeRNAi
c
3 l-LNvs
4 l-LNvs
Percent
d
Pdf mRNA intensity in s-LNvs (A.U.)
***
e
Pdf mRNA intensity in l-LNvs (A.U.)
f
CT2
CT14
Pdf>Dcr2; CD2-HRP
Pdf>Dcr2; poeRNAi, CD2-HRP
g
s-LNvs
l-LNvs
CT5
CT20
Controls
+>poeRNAi
Pdf>Dcr2
Pdf>Dcr2; poeRNAi
PDF
PER
Merge

Supplementary Figure 3. Effects of PDF-specific *poe* knockdown on cell viability, neuronal morphology, and dPER expression in fly clock neurons. a Representative photomicrographs of RNA *in situ* hybridization showing *Pdf* (green) and *poe* (magenta) mRNA expression in the whole fly brain. Scale bar, 60 μm. **b, c** Percentage of *Pdf>Dcr2; poe*^{RNAi} flies and controls containing either 3 or 4 s-LN$_v$s (**b**) or l-LN$_v$s (**c**) per hemisphere. n = 35 flies per genotype. Chi-square test. **d, e** Quantification of fluorescence intensity of *Pdf* ISH signal in the s-LN$_v$s (**d**) or l-LN$_v$s (**e**) of *Pdf>Dcr2; poe*^{RNAi} flies and controls at ZT 8. +>*poe*^{RNAi}: n = 4, *Pdf>Dcr2:* n = 6 (**d**) or 7 (**e**), *Pdf>Dcr2; poe*^{RNAi}: n = 6 (**d**) or 8 (**e**). ***p = 0.0007 as indicated; one-way ANOVA with Bonferroni's post hoc. **f** Representative photomicrographs of CD2-HRP expression at CT 2 and CT 14 on DD3 in the whole brain of *Pdf> Dcr2; CD2-HRP* and *Pdf> Dcr2; poe*^{RNAi}*, CD2-HRP* flies. Scale bar, 100 μm. **g** Representative photomicrographs of dPER (magenta) and PDF (green) immunofluorescence in the s-LN$_v$s (left) and l-LN$_v$s (right) of *Pdf>Dcr2; poe*^{RNAi} flies and controls at CT 5 and CT 20 on DD3. Scale bar, 10 μm. Values represent percent (**b, c**) or mean ± SEM (**d, e**). 'n' represents number of flies. A.U., arbitrary units. Source data are provided as a Source Data file.

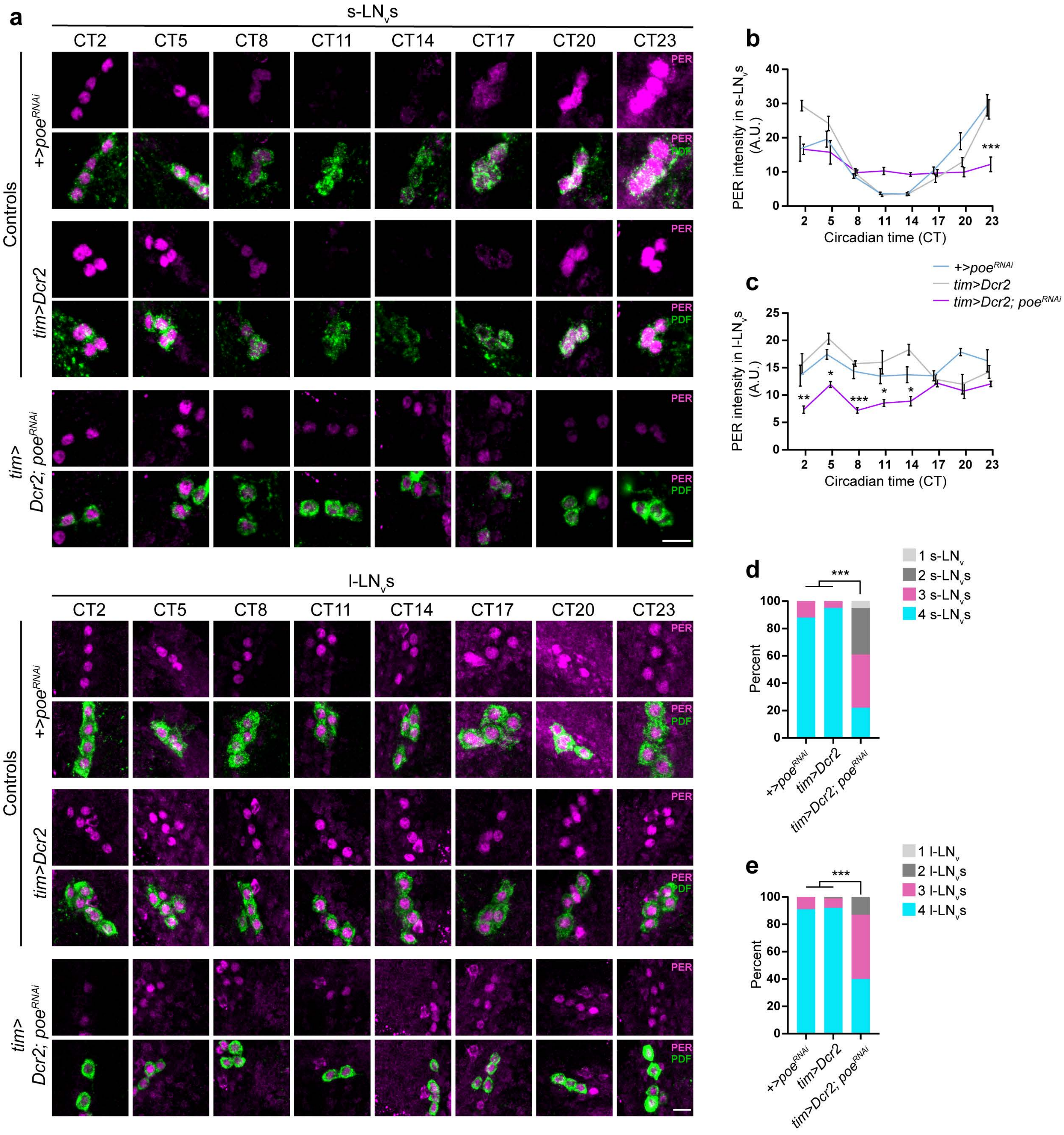

a
s-LNvs
CT2
CT5
CT8
CT11
CT14
CT17
CT20
CT23
Controls
+>poeRNAi
tim>Dcr2
tim> Dcr2; poeRNAi
PER
PDF
l-LNvs
b
PER intensity in s-LNvs (A.U.)
Circadian time (CT)
***
c
PER intensity in l-LNvs (A.U.)
Circadian time (CT)
**
*
***
+>poeRNAi
tim>Dcr2
tim>Dcr2; poeRNAi
d
1 s-LNv
2 s-LNvs
3 s-LNvs
4 s-LNvs
Percent
***
e
1 l-LNv
2 l-LNvs
3 l-LNvs
4 l-LNvs
Percent
***

Supplementary Figure 4. Effects of *tim*-specific *poe* knockdown on dPER expression and cell viability in fly clock neurons. a Representative photomicrographs of dPER (magenta) and PDF (green) immunofluorescence in the s-LN_vs (top) and l-LN_vs (bottom) of *tim>Dcr2; poe*^{RNAi} flies and controls across the circadian cycle on DD3. Scale bar, 10 µm. **b** Quantification of dPER-IR intensity in the s-LN_vs of *tim>Dcr2; poe*^{RNAi} flies and controls. For each fly, a mean intensity value was calculated by taking the average of all s-LN_vs that were present in one hemisphere. In ascending order of CT: n = 9, 9, 8, 7, 9, 10, 10, 9 for +>*poe*^{RNAi}; n = 7, 8, 10, 9, 9, 10, 9, 11 for *Pdf>Dcr2*; n = 6, 6, 8, 8, 6, 8, 8, 7 for *Pdf>Dcr2; poe*^{RNAi}. ***p<0.0001 vs. controls; two-way ANOVA with Bonferroni's post hoc. **c** Quantification of dPER-IR intensity in the l-LN_vs of *tim>Dcr2; poe*^{RNAi} flies and controls, as described above in (**b**). In ascending order of CT: n = 9, 8, 8, 8, 9, 10, 10,10 for +>*poe*^{RNAi}; n = 7, 9, 9, 9, 9, 9, 9, 10 for *Pdf>Dcr2*; n = 6, 5, 9, 8, 6, 7, 9, 9 for *Pdf>Dcr2; poe*^{RNAi}. CT2: **p = 0.0055, CT5: *p = 0.0299, CT8: ***p = 0.0003, CT11: *p = 0.0268, CT14: *p = 0.0429 vs. +>*poe*^{RNAi}*;* two-way ANOVA with Bonferroni's post hoc. **d, e** Percentage of *tim>Dcr2; poe*^{RNAi} flies and controls containing either 1, 2, 3, or 4 s-LN_vs (**d**) or l-LN_vs (**e**) per hemisphere. +>*poe*^{RNAi}: n = 64, *Pdf>Dcr2:* n = 64 (**d**) or 61 (**e**), *Pdf>Dcr2; poe*^{RNAi}: n = 64(**d**) or 62 (**e**). ***p<0.0001 vs. controls; two-sided chi-square test. Values represent mean ± SEM (**b, c**) or percent (**d, e**). 'n' represents number of flies. A.U., arbitrary units. Source data are provided as a Source Data file.

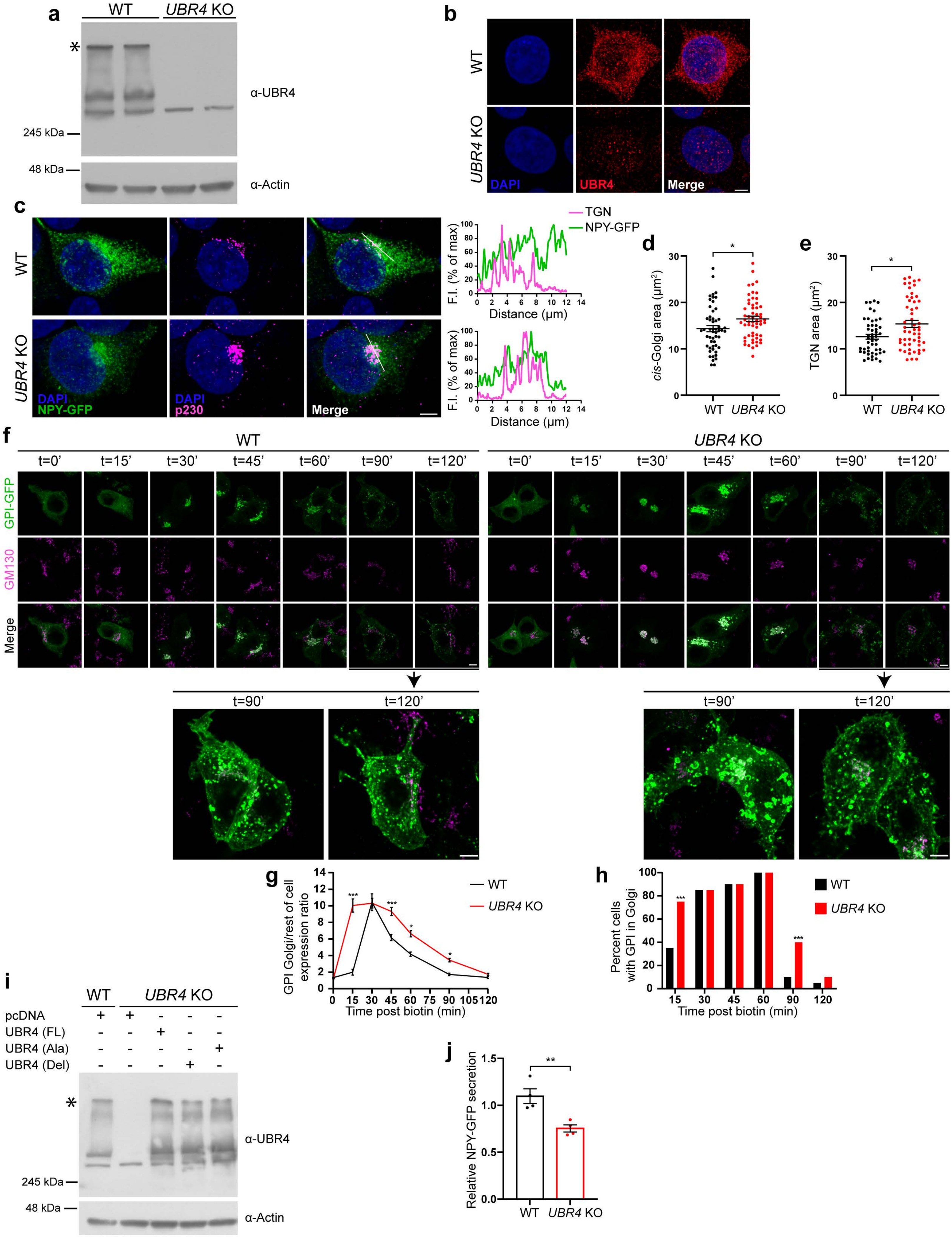

a
WT
UBR4 KO
*
α-UBR4
245 kDa
48 kDa
α-Actin
b
WT
UBR4 KO
DAPI
UBR4
Merge
c
WT
UBR4 KO
DAPI
NPY-GFP
DAPI
p230
Merge
TGN
NPY-GFP
F.I. (% of max)
Distance (μm)
d
cis-Golgi area (μm²)
WT
UBR4 KO
e
TGN area (μm²)
f
GPI-GFP
GM130
Merge
t=0'
t=15'
t=30'
t=45'
t=60'
t=90'
t=120'
g
GPI Golgi/rest of cell expression ratio
Time post biotin (min)
h
Percent cells with GPI in Golgi
Time post biotin (min)
i
pcDNA
UBR4 (FL)
UBR4 (Ala)
UBR4 (Del)
α-UBR4
α-Actin
j
Relative NPY-GFP secretion

Supplementary Figure 5. UBR4 depletion impairs cargo transport along the secretory pathway. a Western blot of UBR4 from *UBR4* WT and KO HEK293T cell lysates. Asterisk denotes the full-length UBR4 polypeptide. **b** Representative photomicrographs showing UBR4 immunofluorescence (red) in *UBR4* WT and KO HEK293T cells. DAPI, blue. **c** Representative photomicrographs of transfected *UBR4* WT and KO HEK293T cells showing immunofluorescence of NPY-GFP (green) and the TGN marker, p230 (magenta). Profile plots (right) show NPY-GFP and p230 fluorescence intensity (F.I.) along the reference axis (white line, merged panel). **d** Area of *cis*-Golgi. WT: n = 53, *UBR4* KO: n = 57. *p = 0.0141; two-tailed Mann-Whitney U test. **e** Area of TGN. WT: n = 49, *UBR4* KO: n = 53. *p = 0.0133; two-tailed Mann-Whitney U test. **f** Representative photomicrographs showing the trafficking of GPI-GFP (green) in *UBR4* WT and KO HEK293T cells using the RUSH assay. Cells were fixed at the indicated time points post-biotin and CHX addition, and immunostained for GPI-GFP and GM130 (magenta). Images for t=90' and t=120' are presented at higher magnification to show the preponderance of large GPI-GFP$^+$ vesicles that appeared in the cytoplasm of *UBR4* KO cells at these time points. **g** Ratio of GPI-GFP abundance in the Golgi relative to the rest of the cell at specified time points post-biotin addition in the RUSH experiment. In ascending order of time post-biotin addition (t): n = 20, 20, 26, 40, 15, 24, 14 for WT; n = 20, 25, 29, 31, 17, 41, 27 for *UBR4* KO. t = 60min: *p = 0.0308, t = 90min: *p = 0.0409, ***p<0.0001 vs. WT; two-way ANOVA with Bonferroni's post hoc. **h** Percentage of cells with GPI-GFP localization in the Golgi of *UBR4* WT and KO HEK293T cells at specified time points post-biotin addition in the RUSH experiment. ***p<0.0001 vs. WT; two-tailed chi-square test. **i** Western blot of UBR4 from *UBR4* WT and KO HEK293T cell lysates transfected with the plasmids: empty vector (pcDNA), UBR4 (FL), UBR4 (Ala), or UBR4 (Del). Asterisk denotes the full-length UBR4 polypeptide. **j** Relative NPY-GFP secretion from *UBR4* WT and KO HEK293T cells assayed via ELISA. n = 4 per genotype. **p = 0.0074; two-tailed unpaired t-test. Values represent mean ± SEM (**d, e, g, j**) or percent (**h**). 'n' represents number of cells (**d, e, g**) or samples (**j**). Scale bar, 5 μm. Source data are provided as a Source Data file.

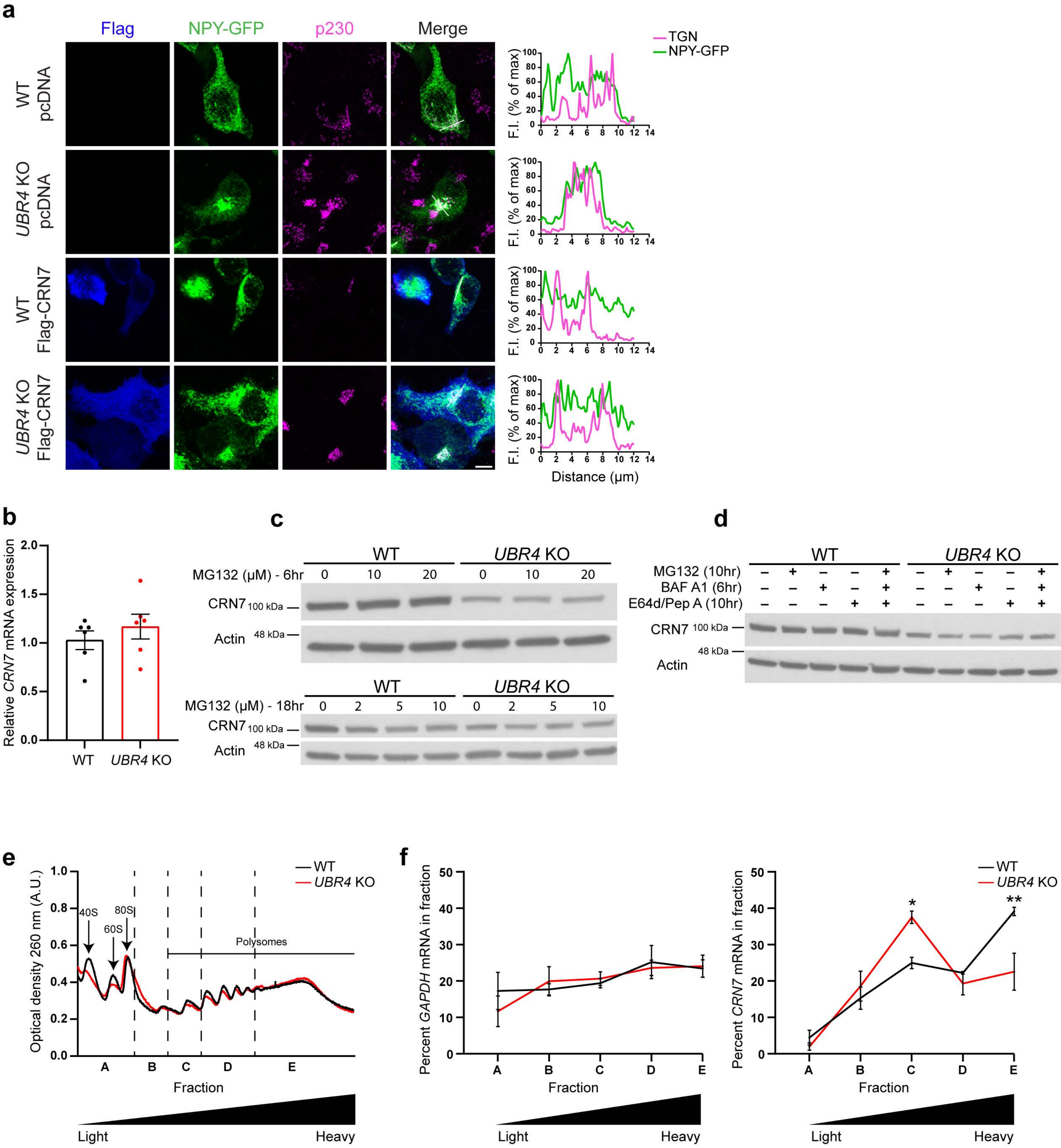

a
Flag
NPY-GFP
p230
Merge
TGN
NPY-GFP
WT pcDNA
UBR4 KO pcDNA
WT Flag-CRN7
UBR4 KO Flag-CRN7
F.I. (% of max)
Distance (μm)
b
Relative CRN7 mRNA expression
WT
UBR4 KO
c
WT
UBR4 KO
MG132 (μM) - 6hr
0
10
20
CRN7
100 kDa
Actin
48 kDa
MG132 (μM) - 18hr
0
2
5
10
d
MG132 (10hr)
BAF A1 (6hr)
E64d/Pep A (10hr)
e
WT
UBR4 KO
Optical density 260 nm (A.U.)
40S
60S
80S
Polysomes
A
B
C
D
E
Fraction
Light
Heavy
f
Percent GAPDH mRNA in fraction
Percent CRN7 mRNA in fraction

Supplementary Figure 6. Overexpression of Coronin 7 rescues the retention of NPY-GFP in the Golgi of *UBR4* KO cells. a Representative photomicrographs of NPY-GFP (green) immunofluorescence in *UBR4* WT and KO HEK293T cells transfected with either pcDNA empty vector or Flag-CRN7 (blue). p230, magenta. Profile plots (right) show NPY-GFP and p230 fluorescence intensity (F.I.) along the reference axis (white line, merged panel). Scale bar, 10 μm. **b** qRT-PCR analysis of relative *CRN7* mRNA expression in untransfected *UBR4* WT and KO HEK293T cells. *GAPDH* was used as the normalization control. n = 6 samples per group; two-tailed unpaired t-test. **c, d** Western blot of endogenous CRN7 following treatment of *UBR4* WT and KO HEK293T cells with vehicle (DMSO, 0μM MG132) or MG132 at the indicated concentrations for 6 hours (top) or 18 hours (bottom) (**c**), or with MG132 (10μM), Bafilomycin A1 (500nM), and/or E64d plus Pepstatin A (10μg/mL each) for the indicated duration (**d**). **e** Polysome profiles of untransfected *UBR4* WT and KO HEK293T cells. Positions of the 40S, 60S, and 80S ribosome peaks and polysomes are indicated. A.U., arbitrary units. **f** qRT-PCR analysis showing levels of *GAPDH* and *CRN7* mRNA extracted from polysome fractions of *UBR4* WT and KO HEK293T cells. n = 3 samples per group. **p* = 0.0233, ***p* = 0.0063 vs. WT; two-way ANOVA. All values represent mean ± SEM. Source data are provided as a Source Data file.

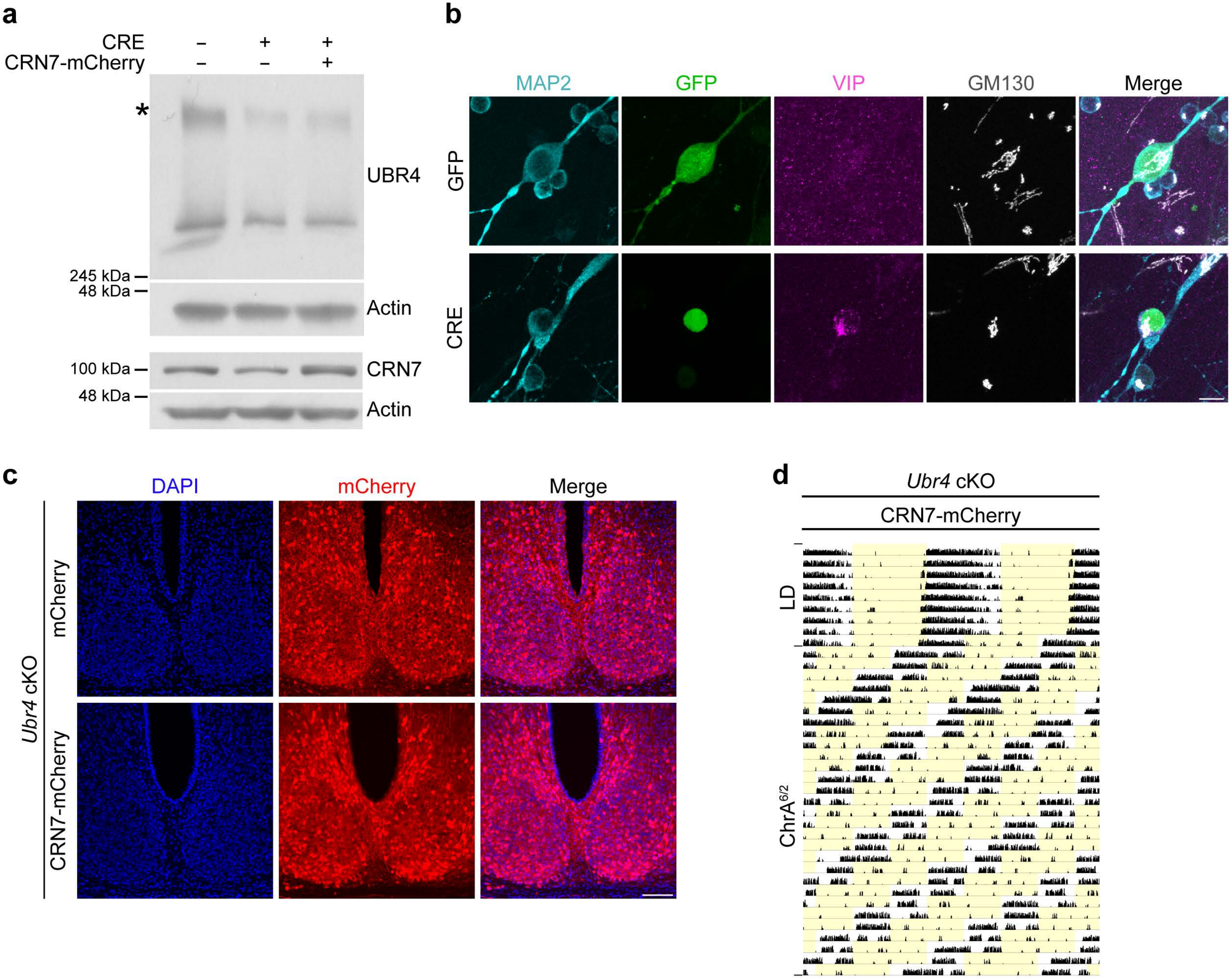
a
CRE
CRN7-mCherry
*
UBR4
245 kDa
48 kDa
Actin
100 kDa
CRN7
48 kDa
Actin
b
MAP2
GFP
VIP
GM130
Merge
GFP
CRE
c
DAPI
mCherry
Merge
Ubr4 cKO
mCherry
CRN7-mCherry
d
Ubr4 cKO
CRN7-mCherry
LD
ChrA6/2

Supplementary Figure 7. The effects of *Ubr4* ablation on VIP trafficking in SCN neuronal cultures and SCN-targeted overexpression of *Crn7* on circadian behavior. a Western blot of UBR4 and CRN7 from *Ubr4*$^{fl/fl}$ SCN neuronal cultures that had been transduced with the indicated AAV1 vectors. Asterisk denotes the full-length UBR4 polypeptide. Similar results were obtained from 2 independent experiments. **b** Representative photomicrographs showing endogenous VIP (magenta) expression in *Ubr4*$^{fl/fl}$ SCN neuronal cultures that had been transduced with AAVs expressing either GFP (AAV1-hSyn-eGFP (GFP)) or Cre-recombinase (AAV1-hSyn-Cre-eGFP (CRE)). Cells were co-immunostained for MAP2 (cyan), GFP (green), and GM130 (gray). Scale bar, 10 µm. Similar results were obtained from 3 independent experiments. **c** Representative photomicrographs of mCherry immunoreactivity (red) in the SCN of *Ubr4* cKO mice following SCN injections of either AAV1-CMV-mCherry (mCherry) or AAV1-CMV-m-Coronin7-2A-mCherry (CRN7-mCherry). DAPI, blue. Scale bar, 100 µm. mCherry: n = 9 mice, CRN7-mCherry: n = 8 mice. **d** A representative double-plotted actogram showing wheel-running activity of an "entrained" *Ubr4* cKO mouse under the chronic jetlag (ChrA$^{6/2}$) paradigm. This animal had received a bilateral injection of AAV1-CMV-m-Coronin7-2A-mCherry (CRN7-mCherry) into the SCN.

Supplementary Table 1. Wheel-running behavioral parameters in control and *Ubr4* cKO mice.

Parameter	Photoperiod	Control		*Ubr4* cKO		P-value
		Mean ± SEM	N	Mean ± SEM	N	
Daily wheel revolutions	LD	5299.28 ± 659.67	11	3482.43 ± 451.81	13	0.0638
	DD	5626.60 ± 733.64	11	4411.69 ± 503.81	12	0.2777
	[a] LL-5 Lux	4163.71 ± 715.99	11	1620.03 ± 247.36	11	<0.0001***
	[a] LL-10 Lux	2672.46 ± 357.56	10	1107.13 ± 120.09	11	0.0062**
	[a] LL-20 Lux	2747.47 ± 448.92	11	825.48 ± 97.62	11	0.0026**
	[a] LL-40 Lux	2602.1 ± 539.25	11	613.76 ± 131.19	11	0.0017**
	[a] LL-80 Lux	2092.22 ± 438.71	11	522.15 ± 141.11	11	0.0227*
	LL-120 Lux	1366.16 ± 299.79	8	437.37 ± 110.57	8	0.0692
	[a] $ChrA^{6/2}$ jetlag	5441.09 ± 276.32	8	2553.16 ± 178.61	11	<0.0001***
	[a] DD- post $ChrA^{6/2}$ jetlag	6196.26 ± 170.09	7	2258.24 ± 248.53	11	<0.0001***
Amplitude (χ^2 Amplitude)	LD	893.71 ± 60.30	11	740.61 ± 32.59	11	0.3410
	DD	1129.73 ± 98.42	11	1078.33 ± 87.77	13	>0.9999
	LL-5 Lux	1210.83 ± 153.65	11	948.11 ± 86.00	11	>0.9999
	LL-10 Lux	1181.53 ± 178.09	11	1230.57 ± 194.78	11	>0.9999
	LL-20 Lux	1073.24 ± 129.64	10	881.09 ± 111.22	10	>0.9999
	[a] LL-40 Lux	1173.55 ± 139.60	10	675.27 ± 67.12	11	0.0277*
	[a] LL-80 Lux	1522.62 ± 195.44	11	650.75 ± 75.89	11	0.0001***
	LL-120 Lux	953.87 ± 138.46	8	677.29 ± 80.02	8	0.6914
	$ChrA^{6/2}$ jetlag	21 hr component<br>1884.50 ± 258.93	9/9	21 hr component<br>1757.50 ± 189.78	13/13	0.6896
		>24 hr component<br>783.70 ± 71.58	5/9	>24 hr component<br>NA	0/13	NA
	[a] DD- post $ChrA^{6/2}$ jetlag	2017.07 ± 110.67	8	1234.37 ± 126.97	12	0.0004***
FFT Amplitude	LD	$1.48 \times 10^{-2} \pm 1.41 \times 10^{-3}$	11	$1.45 \times 10^{-2} \pm 1.20 \times 10^{-3}$	12	>0.9999
	DD	$0.0124 \pm 1.49 \times 10^{-3}$	11	$0.0136 \pm 1.17 \times 10^{-3}$	13	0.9824
	LL-5 Lux	$7.00 \times 10^{-3} \pm 1.00 \times 10^{-3}$	11	$5.00 \times 10^{-3} \pm 6.00 \times 10^{-4}$	11	0.5081
	LL-10 Lux	$6.00 \times 10^{-3} \pm 1.00 \times 10^{-3}$	11	$5.00 \times 10^{-3} \pm 5.00 \times 10^{-4}$	11	0.9097
	LL-20 Lux	$6.00 \times 10^{-3} \pm 1.00 \times 10^{-3}$	11	$4.00 \times 10^{-3} \pm 4.00 \times 10^{-4}$	11	0.3880
	[a] LL-40 Lux	$6.00 \times 10^{-3} \pm 1.00 \times 10^{-3}$	11	$3.00 \times 10^{-3} \pm 3.00 \times 10^{-4}$	11	0.0076**
	LL-80 Lux	$3.00 \times 10^{-3} \pm 4.67 \times 10^{-4}$	10	$2.00 \times 10^{-3} \pm 3.00 \times 10^{-4}$	11	0.2066
	LL-120 Lux	$5.00 \times 10^{-3} \pm 1.00 \times 10^{-3}$	8	$3.00 \times 10^{-3} \pm 5.00 \times 10^{-4}$	8	0.0764
	$ChrA^{6/2}$ jetlag	$4.26 \times 10^{-3} \pm 3.78 \times 10^{-4}$	9	$4.41 \times 10^{-3} \pm 2.28 \times 10^{-4}$	12	>0.9999
	[a] DD- post $ChrA^{6/2}$ jetlag	$7.75 \times 10^{-3} \pm 7.07 \times 10^{-4}$	8	$6.25 \times 10^{-3} \pm 4.15 \times 10^{-4}$	11	0.0327*
[b]Period length (hr)	DD	23.63 ± 0.06	11	23.60 ± 0.04	13	0.6652
	LL-5 Lux	24.85 ± 0.16	11	24.29 ± 0.07	11	0.1160
	[a] LL-10 Lux	25.38 ± 0.20	10	24.37 ± 0.09	11	0.0003***
	[a] LL-20 Lux	25.43 ± 0.20	10	24.67 ± 0.15	9	0.0048**
	[a] LL-40 Lux	25.71 ± 0.22	11	24.63 ± 0.19	10	<0.0001***
	LL-80 Lux	25.8 ± 0.22	11	25.4 ± 0.24	7	>0.9999

Period length (hr) (cont'd)	LL-120 Lux	26.23 ± 0.11	7	25.5 ± 0.19	6	0.8516
	DD- post $ChrA^{6/2}$ jetlag	23.50 ± 0.09	8	23.61± 0.03	12	0.2118
Phase angle	LD	0.35 ± 0.09	10	0.34 ± 0.1	12	0.9493

[a] *p<0.05, **<0.01, ***p<0.001 by linear mixed effects modelling.
[b] excludes all arrhythmic animals in LL.

Supplementary Table 2. Summary of fly locomotor activity parameters using the *tim*-GAL4 driver.

Parameter	Photoperiod	+>poe^{RNAi}		*tim>Dcr2*		*tim> Dcr2;* poe^{RNAi}		P-value
		Mean ± SEM	N	Mean ± SEM	N	Mean ± SEM	N	
Daily locomotor activity counts	LD	435.8 ± 30.33	15	1005 ± 50.72	31	828.4 ± 42.69	31	[b]<0.0001***<br>[c]0.0152*
	DD	378.4 ± 28.24	14	1047 ± 58.05	32	501.1 ± 25.78	30	[b]0.2914<br>[c]<0.0001***
FFT power	LD	0.12 ± $7.46x10^{-3}$	15	0.11 ± 9.32 $x10^{-3}$	32	0.07 ± $4.22x10^{-3}$	30	[b]<0.0001***<br>[c]0.0004***
	DD	0.04 ± $5.3x10^{-3}$	14	0.05 ± $5.3x10^{-3}$	31	0.01 ± $7.20x10^{-4}$	31	[bc]<0.0001***
[a] Period length (h)	DD	23.90 ± 0.03	14	24.17 ± 0.057	29	NA	NA	NA

[a] excludes all arrhythmic flies.
[b] indicates p-value relative to +>poe^{RNAi} control.
[c] indicates p-value relative to *tim>Dcr2* control.
*p<0.05, ***p<0.001 by one-way ANOVA with Bonferroni's post hoc test (LD activity), or Kruskal-Wallis with Dunn's post hoc test (DD activity, FFT power).

Supplementary Table 3. Summary of fly locomotor activity parameters using the *Pdf*-GAL4 driver.

Parameter	Photo-period	+>poe^{RNAi}		*Pdf>Dcr2*		*Pdf> Dcr2;* poe^{RNAi}		P-value
		Mean ± SEM	N	Mean ± SEM	N	Mean ± SEM	N	
[e] Daily locomotor activity counts	LD	361.40 ± 15.55	31	712.2 ± 30.26	30	477 ± 15.74	62	[b]0.0002***<br>[c]<0.0001***
	DD	254.4 ± 14.13	30	591.6 ± 40.68	30	555.5 ± 16.36	104	[b]<0.0001***<br>[c]0.8915
FFT power	[e]LD	0.08 ± $5.95x10^{-3}$	31	0.11 ± $7.18x10^{-3}$	30	0.08 ± $3.56x10^{-3}$	108	[b]>0.9999 [c]0.0133*
	DD	All: 0.032 ± $7.54x10^{-3}$<br>Early: 0.034 ± $2.28x10^{-3}$<br>Late: 0.028 ± $1.89x10^{-3}$	32<br>30<br>30	0.023 ± $1.82x10^{-3}$<br>0.030 ± $2.11x10^{-3}$<br>0.027 ± $2.64x10^{-3}$	29<br>28<br>28	0.012 ± $6.92x10^{-4}$<br>0.035 ± $2.07x10^{-3}$<br>0.011 ± $7.59x10^{-4}$	108<br>104<br>104	[bce]<0.0001***<br>[bd]>0.9999<br>[cd]0.5289,<br>[bcd]<0.0001***
[a] Period length (h)	DD	23.77 ± 0.03	29	24.28 ± 0.03	25	Unstable period	14	NA

[a] excludes all arrhythmic flies.
[b] indicates p-value relative to +>poe^{RNAi} control.
[c] indicates p-value relative to *Pdf>Dcr2* control.
[d] *p<0.05, ***p<0.001 by linear mixed effects modelling with Bonferroni's post hoc test.
[e] *p<0.05, ***p<0.001 by one-way ANOVA with Bonferroni's post hoc test (Activity, FFT power - LD) or Kruskal-Wallis with Dunn's post hoc test (FFT power - DD All).

Supplementary Table 4. Details of Experimental Reagents and Resources.

REAGENT or RESOURCE	SOURCE	IDENTIFIER
Antibodies (and the dilution it was used at)		
Rabbit anti-UBR4 (1:1000)	Abcam	Cat# ab86738; RRID: AB_1952666
Rabbit anti-Actin (1:15K)	Sigma	Cat# A2066; RRID: AB_476693
Rabbit anti-Arginine Vasopressin (AVP) (1:30K)	Sigma	Cat# AB1565; RRID: AB_90782
Rabbit anti-Vasoactive Intestinal Peptide (VIP) (1:1000 and 1:10K)	ImmunoStar	Cat# 20077; RRID: AB_572270
Rabbit anti-PERIOD2 (1:30K)	Gift from D. Weaver	N/A
Rabbit anti-Coronin 7 (1:10K and 1:20K)	Abcam	Cat# ab117446; RRID: AB_10902520
Guinea Pig anti-Cre-Recombinase (1:1000)	Synaptic Systems	Cat# 257004; RRID:AB_2782969
Rabbit anti-FLAG (1:2000)	Abcam	Cat# ab1162; RRID: AB_298215
Mouse anti-GM130 (1:2000)	BD Biosciences	Cat# 610822; RRID: AB_398141
Mouse anti-P230 (1:2000)	BD Biosciences	Cat# 611280; RRID: AB_398808
Chicken anti-MAP2 (1:1000)	Abcam	Cat# ab5392; RRID: AB_2138153
Goat anti-Green Fluorescent Protein (GFP) (1:5000)	Eusera	Cat# EU3
Mouse anti-V5 (1:2000)	Abcam	Cat# ab27671; RRID: AB_471093
Rat anti-mCherry (1:5000)	Thermo Fisher Scientific	Cat# M11217; RRID:AB_2536611
Goat anti-Rabbit IgG Secondary Antibody, HRP conjugate (1:100K)	Thermo Fisher Scientific	Cat# 31460; RRID: AB_228341
Biotinylated goat anti-Rabbit IgG (1:300)	Vector Laboratories	Cat# BA-1000; RRID: AB_2313606
Donkey anti-Goat IgG (H+L) Highly Cross-Adsorbed Secondary Antibody, Alexa Fluor Plus 488 (1:1000)	Thermo Fisher Scientific	Cat# A32814; RRID: AB_2762838
Donkey anti-Rabbit IgG (H+L) Highly Cross-Adsorbed Secondary Antibody, Alexa Fluor 594 (1:1000)	Thermo Fisher Scientific	Cat# A-21207; RRID: AB_141637
Donkey anti-Rabbit IgG (H+L) Highly Cross-Adsorbed Secondary Antibody, Alexa Fluor 488 (1:100)	Thermo Fisher Scientific	Cat# A-21206, RRID:AB_2535792
Donkey anti-Mouse IgG (H+L) Highly Cross-Adsorbed Secondary Antibody, Alexa Fluor 594 (1:1000)	Thermo Fisher Scientific	Cat# A-21203; RRID: AB_2535789
Donkey anti-Mouse IgG (H+L) Highly Cross-Adsorbed Secondary Antibody, Alexa Fluor 647 (1:1000)	Thermo Fisher Scientific	Cat# A-31571, RRID: AB_162542
Donkey anti-Rat IgG (H+L) Highly Cross-Adsorbed Secondary Antibody, Alexa Fluor 594 (1:1000)	Thermo Fisher Scientific	Cat# A-21209, RRID: AB_2535795
DyLight 405 AffiniPure Donkey Anti-Chicken IgY (IgG) (H+L) (1:1000)	Jackson ImmunoResearch Labs	Cat# 703-475-155, RRID: AB_2340373
DyLight 405 AffiniPure Donkey Anti-Guinea Pig IgG (H+L) (1:1000)	Jackson ImmunoResearch Labs	Cat# 706-475-148; RRID: AB_2340470
DyLight 405 AffiniPure Donkey Anti-Rabbit IgG (H+L) (1:1000)	Jackson ImmunoResearch Labs	Cat# 711-475-152; RRID: AB_2340616

Alexa Fluor 594 AffiniPure Donkey Anti-Guinea Pig IgG (H+L) (1:1000)	Jackson ImmunoResearch Labs	Cat# 706-585-148; RRID: AB_2340474
Rabbit anti-Pigment Dispersing Factor (PDF) (1:20K)	Gift from M. Nitabach	N/A
Guinea Pig anti-dPERIOD (GP73) (1:1000)	Gift from I. Edery	N/A
Bacterial and Virus Strains		
One shot TOP10 Chemically Competent *E.coli*	Thermo Fisher Scientific	Cat# C404010
CellLight ™ Golgi-RFP, BacMam 2.0	Thermo Fisher Scientific	Cat# C10593
AAV1-CMV-m-CORO7-2A-mCherry	Vector Biolabs	Cat# AAV-255958
AAV1-CMV-mCherry	Vector Biolabs	Cat# 7103
AAV1-hSyn-mVIP-WPRE	Vector Biolabs	Cat# AAV-276014
pENN.AAV.hSyn.HI.eGFP-Cre.WPRE.SV40 (AAV1)	Addgene (J.M. Wilson)	Addgene viral prep #105540-AAV1
pAAV.hSyn.eGFP.WPRE.bGH (AAV1)	Addgene (J.M. Wilson)	Addgene viral prep # 105539-AAV1
pENN.AAV.hSyn.Cre.WPRE.hGH (AAV1)	Addgene (J.M. Wilson)	Addgene viral prep # 105553-AAV1
Chemicals, Peptides, and Recombinant Proteins		
Protease Inhibitor Cocktail	Sigma-Aldrich	Cat# SRE0055
4-20% Mini-PROTEAN TGX Precast Gels (10/12 wells)	BioRad	Cat# 4561095
Polyvinylidene difluoride (PVDF) membranes	Millipore	Cat# IPVH00010
SuperSignal West Femto Maximum Sensitivity Substrate	Thermo Fisher Scientific	Cat# 34095
VECTASTAIN ABC-HRP kit	Vector Laboratories	Cat# PK-4000
DAB Peroxidase (HRP) Substrate Kit (with nickel) 3,3′-diaminobenzidine	Vector Laboratories	Cat# SK-4100
Permount Mounting Media	Thermo Fisher Scientific	Cat# SP15-500
VECTASHIELD Antifade Mounting Medium	Vector Laboratories	Cat# H-1000
iTaq Universal SYBR Green Supermix	BioRad	Cat# 1725124
SsoFast EvaGreen Supermix	BioRad	Cat# 1725201
Fetal Bovine Serum (FBS), Premium	Wisent	Cat# 098150
Penicillin-Streptomycin (10,000 U/mL)	Thermo Fisher Scientific	Cat# 15140-122
0.25% Trypsin/EDTA	Wisent	Cat# 325-043-EL
Poly-D-lysine	Sigma	Cat# P6403
Lipofectamine 3000 Transfection Reagent	Thermo Fisher Scientific	Cat# L3000015
D-Biotin	Bio Basic	Cat# BB0078
Cycloheximide	Sigma	Cat# C7698
MG-132	Sigma	Cat# 474787
Bafilomycin A1	Cayman Chemical	Cat# 11038-500
E64d	Selleck Chemicals	Cat# S7393
Pepstatin A	Tocris Bioscience	Cat# 1190/10
Sodium pyruvate (100mM)	Thermo Fisher Scientific	Cat# 11360070

HEPES (1M)	Thermo Fisher Scientific	Cat#15630080
L-Glutamine	Thermo Fisher Scientific	Cat# 25030081
B-27 supplement	Thermo Fisher Scientific	Cat# 17504044
Critical Commercial Assays		
RNAscope Multiplex Fluorescent Reagent Kit v2	Advanced Cell Diagnostics	Cat# 323100
RNeasy Micro Kit	QIAGEN	Cat# 74004
Presto Endotoxin Free Mini Plasmid Kit	Geneaid	Cat# PEH100
PureYield Plasmid Midiprep System	Promega	Cat# A2492
iScript cDNA synthesis kit	BioRad	Cat# 1708890
GFP ELISA kit	Abcam	Cat# ab171581
mCherry ELISA kit	Abcam	Cat# ab221829
ProteoExtract kit	MillliporeSigma	Cat# 5391801KIT
Deposited Data		
MS proteomics data	PRIDE repository	Project accession: PXD020630
Experimental Models: Cell Lines		
Human: WT HEK293T cells	Gift from S.Tripathi [1]	N/A
Human: *UBR4* KO HEK293T cells	Gift from S.Tripathi [1]	N/A
Experimental Models: Organisms/Strains		
Mouse: C57BL6/J	The Jackson Laboratory	JAX: 000664
Mouse: *Slc32a1*$^{tm2(cre)Lowl}$ (*Vgat-ires-Cre)*	The Jackson Laboratory [2]	JAX: 016962
Mouse: *Ubr4*$^{tm1.2Nkt}$ (*p600* $^{flox/flox}$)	The Jackson Laboratory [3]	JAX: 024844
D.melanogaster: w^{1118}	Bloomington	RRID: BDSC_5905
D.melanogaster: UAS-*Dicer2; tim*-GAL4	Gift from O. Shafer	N/A
D. melanogaster: *Pdf*-GAL4	Gift from P. Taghert	N/A
D. melanogaster: *elav [C155];* UAS-*Dicer2*	Bloomington	RRID: BDSC_25750
D. melanogaster: UAS-*Dicer2*	Bloomington	RRID: BDSC_24651
D. melanogaster: UAS-*poe RNAi*; P{KK101471}VIE-260B	Vienna Drosophila Resource Center	VDRC: 108296; Flybase ID: FBgn0011230
D. melanogaster: UAS-*CD2-HRP*	C.-H. Lee	N/A
Oligonucleotides		
qPCR primers, See Supplementary Table 5	IDT	N/A
NPY cloning primers, See Supplementary Table 5	IDT	N/A
ON-TARGETplus Mouse *Ubr4* siRNA, SMARTpool	Horizon Discovery	Cat# L-050850-00-0005
ON-TARGETplus non-targeting pool	Horizon Discovery	Cat# D-001810-10-20
Recombinant DNA		
NPY-EGFP	Addgene [4]	Addgene plasmid #74629
pCAGImC_Empty	Addgene [5]	Addgene plasmid # 92015
pmCherry-C1 mCherry-NLS	Addgene [6]	Addgene plasmid #58476
Str-KDEL_SBP-EGFP-GPI	Addgene [7]	Addgene plasmid #65294
Str-KDEL_SBP-EGFP-NPY	This paper	N/A

Flag-CORONIN 7	Gift from R.-H. Chen [8]	N/A
V5-PER2	Gift from N. Cermakian	N/A
V5-hUbr4-IRES-mCherry	This paper	N/A
V5-hUbr4 (Ala)-IRES-mCherry	This paper	N/A
V5-hUbr4 (Del)-IRES-mCherry	This paper	N/A
Software and Algorithms		
ClockLab Software	Actimetrics	http://www.actimetrics.com/products/clocklab/
ImageJ 1.52a	National Institute of Health, USA	https://imagej.nih.gov/ij/
Metamorph v7.10	Molecular Devices	https://www.moleculardevices.com/products/cellular-imaging-systems/acquisition-and-analysis-software/metamorph-microscopy#gref
Zen 2010 Software	ZEISS	https://www.zeiss.com/corporate/int/home.html
MaxQuant v1.6.6.0	See reference [9]	https://maxquant.org/
Perseus v1.6.6.0	See reference [10]	https://maxquant.org/perseus
DAVID Bioinformatics Resources 6.8	See reference [11,12]	https://david.ncifcrf.gov/tools.jsp
DAM system	TriKinetics Inc.	https://trikinetics.com/
MATLAB R2020a	Mathworks, Inc.	https://www.mathworks.com/products/matlab.html
Fly toolbox	Levine lab [13]	N/A
GraphPad Prism v8.3.1	GraphPad Software	https://www.graphpad.com/
Other		
RNAscope probe: Mm-*Ubr4*	Advanced Cell Diagnostics	Cat# 415971
RNAscope probe: Dm-*poe*	Advanced Cell Diagnostics	Cat# 583491
RNAscope probe: Dm-*Pdf*-C3	Advanced Cell Diagnostics	Cat# 457471-C3
Opal 520 reagent	PerkinElmer Inc	Cat# FP1487A
Opal 570 reagent	PerkinElmer Inc	Cat# FP1488A
C-18 ZipTip	Millipore	Cat# ZTC18S960
Acclaim PepMap trap column	Thermo Fisher Scientific	Cat# 164946
EASY-Spray PepMap analytical column	Thermo Fisher Scientific	Cat# ES803A

Supplementary Table 5. Primer and synthesized DNA sequences for qRT-PCR and cloning.

Primers	
mPer1 [14]	
Forward: TGGCTCAAGTGGCAATGAGTC	Reverse: GGCTCGAGCTGACTGTTCACT
mPer2 [14]	
Forward: CCATCCACAAGAAGATCCTAC	Reverse: GCTCCACGGGTTGATGAAGC
Clock [15]	
Forward: TGTCTCAAGCTGCAAATTTACCA	Reverse: TTTAGATGCTGCATGGCTCCTA
Bmal1 [15]	
Forward: CCGTGCTAAGGATGGCTGTT	Reverse: TTGGCTTGTAGTTTGCTTCTG
Avp [15]	
Forward: GCTGCCAGGAGGAGAACTAC	Reverse: AAAAACCGTCGTGGCACTC
Vip [15]	
Forward: CAGTTCCTGGCATTCCTGAT	Reverse: GGTCACCTGCTCCTTCAAAC
Mouse Gapdh (this paper)	
Forward: CATGGCCTTCCGTGTTCCTA	Reverse: CCTGCTTCACCACCTTCTTGA
Human GAPDH (this paper)	
Forward: CCATGGGGAAGGTGAAGGTC	Reverse: TGAAGGGGTCATTGATGGCA
Human Coronin 7 (this paper)	
Forward: GTGACATTCGAGCAGGAACC	Reverse: CTTGTCCTCTCCTTGGCCTT
poe (this paper)	
Forward: CCAGGTCCCAGTGGCGTTGC	Reverse: AGGCGCACCTCATTGCCAGG
rp49 [16]	
Forward: ATCGGTTACGGATCGAACAA	Reverse: GACAATCTCCTTGCGCTTCT
NPY cloning primers (this paper)	
Forward: AGGGCCGGCCATACCCCTCCAAGCCGGACAAC	
Reverse: AGCTCGAGTTACCACATTGCAGGGTCTTCAAG	

Gateway LR Cloning			
Entry clone	Destination vector	Final construct	Expressed protein
pENTR3ChUBR4	pCAGImC_Empty	pCAGImC_hUbr4	V5-hUbr4-IRES-mCherry
pENTR3C_hUBR4_ubrbox_Ala	pCAGImC_Empty	pCAGImC_hUbr4(Ala)	V5-hUbr4 (Ala)-IRES-mCherry
pENTR3C_hUBR4_ubrbox_Del	pCAGImC_Empty	pCAGImC_hUbr4(Del)	V5-hUbr4 (Del)-IRES-mCherry

Synthesized DNA for construction of pENTR3C_hUBR4_ubrbox_Ala plasmid (this paper)

ggtaccacgctcacaccgctaaaatggtggatggcgtgggtgtcgccacagtggctgctaaggtgtgcgccaaggatgctgagatttcctatgccaagtatggatc
cttcttcgctgacgctggagccaaggaagatggcagcgctttggctctggtgaagagaactcctagcagtggcatgagctctaccatgaaggagtcggcatttcag
agtgaacccaggatttcagagagtctagtgcgtcatgccagcacctcctcgccagctgacaaagccaaggttaccatcagtgatggaaaggttgctgacgaaga
gaagcccaagaagagcagcctctgccgcacagtagagggctgccgggaggaattacagaaccaggccaatttctccttcgctcctctcgtgttagacatgcttaa
tttccttatggatgccattcagaccaacttccagcaagcttcagccgtcgggagcagcagccgtgctcagcaagccctcagtgagctacacactgtggagaaggc
agtggagatgacagaccagctgatggttcccaccttagggtcccaggaaggtgcctttgagaatgtgcggatgaattacagtggagaccagggccagaccatcc
ggcagctgatcagtgctcatgtgctcaggcgggtggctatgtgtgtgctctcctctccccatgggcgccgccaacatttggctgtcagccatgagaagggcaagatc
accgttctgcagctctctgcactcctgaagcaagcagattccagcaaaaggaagttaactctgacccgcttggcttctgccccagttccttttactgtgttgagcctcac
aggaaatccctgcaaggaagactacttggcggtttgtgggctaaaggactgtcatgtgctcacctttagtagctcaggctctgtttcggatcacttggttttgcaccctca
gttggcaacggggaacttcatcatcaaagccgtgtggttacctggt

Synthesized DNA for construction of pENTR3C_hUBR4_ubrbox _Del plasmid (this paper)

ggtaccacgtgaagagaactcctagcagtggcatgagctctaccatgaaggagtcggcatttcagagtgaacccaggatttcagagagtctagtgcgtcatgcca
gcacctcctcgccagctgacaaagccaaggttaccatcagtgatggaaaggttgctgacgaagagaagcccaagaagagcagcctctgccgcacagtagag
ggctgccgggaggaattacagaaccaggccaatttctccttcgctcctctcgtgttagacatgcttaatttccttatggatgccattcagaccaacttccagcaagcttc
agccgtcgggagcagcagccgtgctcagcaagccctcagtgagctacacactgtggagaaggcagtggagatgacagaccagctgatggttcccaccttagg
gtcccaggaaggtgcctttgagaatgtgcggatgaattacagtggagaccagggccagaccatccggcagctgatcagtgctcatgtgctcaggcgggtggctat
gtgtgtgctctcctctccccatgggcgccgccaacatttggctgtcagccatgagaagggcaagatcaccgttctgcagctctctgcactcctgaagcaagcagatt
ccagcaaaaggaagttaactctgacccgcttggcttctgccccagttccttttactgtgttgagcctcacaggaaatccctgcaaggaagactacttggcggtttgtgg
gctaaaggactgtcatgtgctcacctttagtagctcaggctctgtttcggatcacttggttttgcaccctcagttggcaacggggaacttcatcatcaaagccgtgtggtt
acctggt

Supplementary References

1. Tripathi, S. *et al.* Meta- and Orthogonal Integration of Influenza 'oMICs' Data Defines a Role for UBR4 in Virus Budding. *Cell Host Microbe* **18**, 723–735 (2015).

2. Vong, L. *et al.* Leptin Action on GABAergic Neurons Prevents Obesity and Reduces Inhibitory Tone to POMC Neurons. *Neuron* **71**, 142–154 (2011).

3. Nakaya, T. *et al.* p600 Plays Essential Roles in Fetal Development. *PLoS One* **8**, e66269 (2013).

4. Taraska, J. W., Perrais, D., Ohara-Imaizumi, M., Nagamatsu, S. & Almers, W. Secretory granules are recaptured largely intact after stimulated exocytosis in cultured endocrine cells. *Proc. Natl. Acad. Sci. U. S. A.* **100**, 2070–2075 (2003).

5. Golden, R. J. *et al.* An Argonaute phosphorylation cycle promotes microRNA-mediated silencing. *Nature* **542**, 197–202 (2017).

6. Belin, B. J., Lee, T. & Mullins, R. D. DNA damage induces nuclear actin filament assembly by Formin -2 and Spire-½ that promotes efficient DNA repair. *Elife* **4**, e07735 (2015).

7. Boncompain, G. *et al.* Synchronization of secretory protein traffic in populations of cells. *Nat. Methods* **9**, 493–498 (2012).

8. Yuan, W. C. *et al.* K33-Linked Polyubiquitination of Coronin 7 by Cul3-KLHL20 Ubiquitin E3 Ligase Regulates Protein Trafficking. *Mol. Cell* **54**, 586–600 (2014).

9. Cox, J. & Mann, M. MaxQuant enables high peptide identification rates, individualized p.p.b.-range mass accuracies and proteome-wide protein quantification. *Nat. Biotechnol.* **26**, 1367–1372 (2008).

10. Tyanova, S. *et al.* The Perseus computational platform for comprehensive analysis of (prote)omics data. *Nature Methods* **13**, 731–740 (2016).

11. Huang, D. W., Sherman, B. T. & Lempicki, R. A. Bioinformatics enrichment tools: Paths toward the comprehensive functional analysis of large gene lists. *Nucleic Acids Res.* **37**, 1–13 (2009).

12. Huang, D. W., Sherman, B. T. & Lempicki, R. A. Systematic and integrative analysis of large gene lists using DAVID bioinformatics resources. *Nat. Protoc.* **4**, 44–57 (2009).

13. Levine, J. D., Funes, P., Dowse, H. B. & Hall, J. C. Signal analysis of behavioral and molecular cycles. *BMC Neurosci.* **3**, 1 (2002).

14. Fustin, J.-M. *et al.* Rhythmic Nucleotide Synthesis in the Liver: Temporal Segregation of Metabolites. *Cell Rep.* **1**, 341–349 (2012).

15. Parsons, M. J. *et al.* The Regulatory Factor ZFHX3 Modifies Circadian Function in SCN via an at Motif-Driven Axis. *Cell* **162**, 607–621 (2015).

16. Krupp, J. J. *et al.* Pigment-Dispersing Factor Modulates Pheromone Production in Clock Cells that Influence Mating in Drosophila. *Neuron* **79**, 54–68 (2013).
