# Supplementary material for: UBR4/POE facilitates secretory trafficking to maintain circadian clock synchrony
Source: Nat Commun. 2022 Mar 24;13:1594. doi: 10.1038/s41467-022-29244-1 (PMC8948264; doi:10.1038/s41467-022-29244-1)
Supplement: Supplementary file 3 — Description of Additional Supplementary Files [file 41467_2022_29244_MOESM3_ESM.pdf]

Description of Additional Supplementary Files

File name: Supplementary Data 1

Description: **List of differentially expressed proteins between *UBR4* WT and KO HEK293T cells.**

Protein expression is represented as Log2 transformed label-free quantification (LFQ) values. Data were analyzed for differential expression using Perseus via a two-tailed student‟s t-test (BH.q FDR < 0.05).

File name: Supplementary Data 2

Description: **FAT Gene Ontology enrichment analysis of the up- and down-regulated proteins in *UBR4* KO HEK293T cells.**

Data were analyzed using DAVID via Fisher‟s exact test (p-value < 0.05). BP is biological process, MF is molecular function, and CC is cellular component.

File name: Supplementary Movie 1

Description: **Time-lapse imaging of NPY-GFP (green) trafficking in *UBR4* WT and KO HEK293T cells in the RUSH experiment.**

The Golgi (magenta) was labeled with Golgi-RFP BacMam 2.0. Select images from this movie were used to generate Fig. 6j.
