# Supplementary material for: UBR4/POE facilitates secretory trafficking to maintain circadian clock synchrony
Source: Nat Commun. 2022 Mar 24;13:1594. doi: 10.1038/s41467-022-29244-1 (PMC8948264; doi:10.1038/s41467-022-29244-1)
Supplement: Supplementary file 7 — Reporting Summary [file 41467_2022_29244_MOESM7_ESM.pdf]

nature research

Corresponding author(s): Hai-Ying Mary Cheng & Joel Levine

Last updated by author(s): February 4, 2022

# Reporting Summary

Nature Research wishes to improve the reproducibility of the work that we publish. This form provides structure for consistency and transparency in reporting. For further information on Nature Research policies, see our Editorial Policies and the Editorial Policy Checklist.

## Statistics

For all statistical analyses, confirm that the following items are present in the figure legend, table legend, main text, or Methods section.

| n/a | Confirmed | |
|---|---|---|
| ☐ | ☒ | The exact sample size (*n*) for each experimental group/condition, given as a discrete number and unit of measurement |
| ☐ | ☒ | A statement on whether measurements were taken from distinct samples or whether the same sample was measured repeatedly |
| ☐ | ☒ | The statistical test(s) used AND whether they are one- or two-sided<br>*Only common tests should be described solely by name; describe more complex techniques in the Methods section.* |
| ☒ | ☐ | A description of all covariates tested |
| ☐ | ☒ | A description of any assumptions or corrections, such as tests of normality and adjustment for multiple comparisons |
| ☐ | ☒ | A full description of the statistical parameters including central tendency (e.g. means) or other basic estimates (e.g. regression coefficient) AND variation (e.g. standard deviation) or associated estimates of uncertainty (e.g. confidence intervals) |
| ☐ | ☒ | For null hypothesis testing, the test statistic (e.g. *F*, *t*, *r*) with confidence intervals, effect sizes, degrees of freedom and *P* value noted<br>*Give P values as exact values whenever suitable.* |
| ☒ | ☐ | For Bayesian analysis, information on the choice of priors and Markov chain Monte Carlo settings |
| ☒ | ☐ | For hierarchical and complex designs, identification of the appropriate level for tests and full reporting of outcomes |
| ☒ | ☐ | Estimates of effect sizes (e.g. Cohen's *d*, Pearson's *r*), indicating how they were calculated |

*Our web collection on statistics for biologists contains articles on many of the points above.*

## Software and code

Policy information about availability of computer code

| | |
|---|---|
| Data collection | ClockLab Software 6.0.30, DAM system. |
| Data analysis | ClockLab Software 6.0.30, ImageJ 1.52a, Metamorph v7.10, Zen 2010 Software, MaxQuant v1.6.6.0, Perseus v1.6.6.0, DAVID Bioinformatics Resources 6.8, MATLAB R2020a, GraphPad Prism v8.3.1, Xcalibur software 4.1.31.9 |

For manuscripts utilizing custom algorithms or software that are central to the research but not yet described in published literature, software must be made available to editors and reviewers. We strongly encourage code deposition in a community repository (e.g. GitHub). See the Nature Research guidelines for submitting code & software for further information.

## Data

Policy information about availability of data

All manuscripts must include a data availability statement. This statement should provide the following information, where applicable:

- Accession codes, unique identifiers, or web links for publicly available datasets
- A list of figures that have associated raw data
- A description of any restrictions on data availability

The mass spectrometry proteomics data have been deposited to the ProteomeXchange Consortium (http://proteomecentral.proteomexchange.org) via the PRIDE partner repository with the dataset identifier PXD020630 [https://www.ebi.ac.uk/pride/archive/projects/PXD020630]. The human UniProt database used in this study is publicly available at https://www.uniprot.org/help/uniprotkb. All data generated during this study that support our findings are available within the article and its supplementary information files. Further information and requests for data, resources and reagents should be directed to and will be fulfilled by the corresponding authors. Source data are provided with this paper.

# Field-specific reporting

Please select the one below that is the best fit for your research. If you are not sure, read the appropriate sections before making your selection.

☒ Life sciences ☐ Behavioural & social sciences ☐ Ecological, evolutionary & environmental sciences

For a reference copy of the document with all sections, see nature.com/documents/nr-reporting-summary-flat.pdf

# Life sciences study design

All studies must disclose on these points even when the disclosure is negative.

| | |
|---|---|
| Sample size | Sample sizes used in this study were informed by previous studies performed in the investigators' laboratories or by other previously published studies that have used similar methods. Sample citations include:<br><br>Mendoza-Viveros L et al. miR-132/212 Modulates Seasonal Adaptation and Dendritic Morphology of the Central Circadian Clock. Cell Rep. 2017 Apr 18;19(3):505-520.<br>Cheng AH et al. SOX2-Dependent Transcription in Clock Neurons Promotes the Robustness of the Central Circadian Pacemaker. Cell Rep. 2019 Mar 19;26(12):3191-3202.e8.<br>Delventhal R et al. Dissection of central clock function in Drosophila through cell-specific CRISPR-mediated clock gene disruption. Elife. 2019 Oct 15;8:e48308. |
| Data exclusions | Outliers were removed from all analyses presented in this paper. Values were considered outliers if they deviated from the group mean by 2SDs or more. For behavioral analyses, mice that were arrhythmic under LL were excluded from period measurements. Similarly, arrhythmic flies were also excluded from period measurements (pre-established criteria). Behavioral period can only be measured for animals exhibiting a periodic/rhythmic pattern of activity, which is absent in arrhythmic animals, and therefore period is irrelevant in such cases. |
| Replication | Every experiment reported in this study was replicated using multiple subjects (e.g., mice, flies, different batches of cultured HEK293T or Neuro-2a cells) that displayed a consistent phenotype/effect within a group. Across the study, experiments were replicated at least two times and all attempts at replication were successful. |
| Randomization | Experimental subjects (mice, flies and cell lines) of the appropriate genotype, age (if applicable), and sex (if applicable) were randomly allocated to experimental groups for testing. For experiments utilizing both male and female subjects, individuals of each sex were evenly divided amongst groups. |
| Blinding | For behavioral experiments, investigators were not blinded to group allocation during data collection, as the data were acquired by an automated software and were not acquired manually by the investigators. Behavioral analyses that required visual assessment of the data rather than automated software analysis methods were performed by investigators who were blind to group allocation. For all other experiments, data collection (in the case of image acquisition) and data analysis were performed by investigators who were blind to genotype or group allocation. |

# Reporting for specific materials, systems and methods

We require information from authors about some types of materials, experimental systems and methods used in many studies. Here, indicate whether each material, system or method listed is relevant to your study. If you are not sure if a list item applies to your research, read the appropriate section before selecting a response.

## Materials & experimental systems

| n/a | Involved in the study | |
|---|---|---|
| ☐ | ☒ | Antibodies |
| ☐ | ☒ | Eukaryotic cell lines |
| ☒ | ☐ | Palaeontology and archaeology |
| ☐ | ☒ | Animals and other organisms |
| ☒ | ☐ | Human research participants |
| ☒ | ☐ | Clinical data |
| ☒ | ☐ | Dual use research of concern |

## Methods

| n/a | Involved in the study | |
|---|---|---|
| ☒ | ☐ | ChIP-seq |
| ☒ | ☐ | Flow cytometry |
| ☒ | ☐ | MRI-based neuroimaging |

# Antibodies

| | |
|---|---|
| Antibodies used | Primary antibodies:<br><br>Rabbit anti-UBR4, Abcam, Cat# ab86738<br>Rabbit anti-Actin, Sigma, Cat# A2066<br>Rabbit anti-Arginine Vasopressin (AVP), Sigma, Cat# AB1565<br>Rabbit anti-Vasoactive Intestinal Peptide (VIP), ImmunoStar, Cat# 20077 |

Rabbit anti-Coronin 7, Abcam, Cat# ab117446
Guinea Pig anti-Cre-Recombinase, Synaptic Systems, Cat# 257004
Rabbit anti-FLAG, Abcam, Cat# ab1162
Mouse anti-GM130 Clone 35/GM130, BD Biosciences, Cat# 610822
Mouse anti-P230 Clone 15/p230 trans Golgi, BD Biosciences, Cat# 611280
Chicken anti-MAP2, abcam, Cat# ab5392
Goat anti-Green Fluorescent Protein (GFP), Eusera, Cat# EU3
Mouse anti-V5 clone SV5-Pk1, Abcam, Cat# ab27671
Rat anti-mCherry clone 16D7, Thermo Fisher Scientific, Cat# M11217
Rabbit anti-PERIOD2, Gift from D. Weaver
Rabbit anti-Pigment Dispersing Factor (PDF), Gift from M. Nitabach
Guinea Pig anti-dPERIOD (GP73), Gift from I. Edery

Secondary antibodies:
Goat anti-Rabbit IgG Secondary Antibody, HRP conjugate, Thermo Fisher Scientific, Cat# 31460
Biotinylated goat anti-Rabbit IgG, Vector Laboratories, Cat# BA-1000
Donkey anti-Goat IgG (H+L) Highly Cross-Adsorbed Secondary Antibody, Alexa Fluor Plus 488, Thermo Fisher Scientific, Cat# A32814
Donkey anti-Rabbit IgG (H+L) Highly Cross-Adsorbed Secondary Antibody, Alexa Fluor 594, Thermo Fisher Scientific, Cat# A-21207
Donkey anti-Rabbit IgG (H+L) Highly Cross-Adsorbed Secondary Antibody, Alexa Fluor 488, Thermo Fisher Scientific, Cat# A-21206
Donkey anti-Mouse IgG (H+L) Highly Cross-Adsorbed Secondary Antibody, Alexa Fluor 594, Thermo Fisher Scientific, Cat# A-21203
Donkey anti-Mouse IgG (H+L) Highly Cross-Adsorbed Secondary Antibody, Alexa Fluor 647, Thermo Fisher Scientific, Cat# A-31571
Donkey anti-Rat IgG (H+L) Highly Cross-Adsorbed Secondary Antibody, Alexa Fluor 594, Thermo Fisher Scientific, Cat# A-21209
DyLight 405 AffiniPure Donkey Anti-Chicken IgY (IgG) (H+L), Jackson ImmunoResearch Labs, Cat# 703-475-155
DyLight 405 AffiniPure Donkey Anti-Guinea Pig IgG (H+L), Jackson ImmunoResearch Labs, Cat# 706-475-148
DyLight 405 AffiniPure Donkey Anti-Rabbit IgG (H+L), Jackson ImmunoResearch Labs, Cat# 711-475-152
Alexa Fluor 594 AffiniPure Donkey Anti-Guinea Pig IgG (H+L), Jackson ImmunoResearch Labs, Cat# 706-585-148

Validation

All commercial antibodies used in this study have been previously tested and validated by the manufacturer as outlined on the respective manufacturer's website. Most of these antibodies are also well characterized in the literature (see references on manufacturer's website). Listed below is the link to all commercial primary antibodies on the vendor's website, or specific publication/validation details for gifted antibodies:

Rabbit anti-UBR4, Cat# ab86738:
https://www.abcam.com/ubr4p600-antibody-ab86738.html
This antibody was also validated in our study using ubr4 knockout mouse SCN and HEK293 cells.

Rabbit anti-Actin, Sigma, Cat# A2066:
https://www.sigmaaldrich.com/catalog/product/sigma/a2066?lang=en&region=CA

Rabbit anti-Arginine Vasopressin (AVP), Sigma, Cat# AB1565:
https://www.sigmaaldrich.com/catalog/product/mm/ab1565?lang=en&region=CA

Rabbit anti-Vasoactive Intestinal Peptide (VIP), ImmunoStar, Cat# 20077:
https://www.immunostar.com/shop/antibody-catalog/vip-vasoactive-intestinal-peptide-antibody/

Rabbit anti-Coronin 7, Abcam, Cat# ab117446:
https://www.abcam.com/coronin-7-antibody-ab117446.html

Guinea Pig anti-Cre-Recombinase, Synaptic Systems, Cat# 257004
https://sysy.com/product/257004

Rabbit anti-FLAG, Abcam, Cat# ab1162:
https://www.abcam.com/ddddk-tag-binds-to-flag-tag-sequence-antibody-ab1162.html

Mouse anti-GM130 Clone 35/GM130, BD Biosciences, Cat# 610822:
https://www.bdbiosciences.com/us/reagents/research/antibodies-buffers/cell-biology-reagents/cell-biology-antibodies/purified-mouse-anti-gm130-35gm130/p/610822

Mouse anti-P230 Clone 15/p230 trans Golgi, BD Biosciences, Cat# 611280:
https://www.bdbiosciences.com/us/reagents/research/antibodies-buffers/cell-biology-reagents/cell-biology-antibodies/purified-mouse-anti-human-p230-trans-golgi-15p230-trans-golgi/p/611280

Chicken anti-MAP2, abcam, Cat# ab5392
https://www.abcam.com/map2-antibody-ab5392.html

Goat anti-Green Fluorescent Protein (GFP), Eusera, Cat# EU3:
http://www.eusera.com/products.htm

Mouse anti-V5 clone SV5-Pk1, Abcam, Cat# ab27671:
https://www.abcam.com/v5-tag-antibody-sv5-pk1-ab27671.html

Rat anti-mCherry clone 16D7, Thermo Fisher Scientific, Cat# M11217
https://www.thermofisher.com/antibody/product/mCherry-Antibody-clone-16D7-Monoclonal/M11217

Rabbit anti-PERIOD2, Gift from D. Weaver:

Control mouse brain tissues stained with this antibody displayed the expected temporal and spatial expression of the mPER2 protein in line with previous literature. Also, this antibody was previously validated/used in:
LeSauter, J., Lambert, C.M., Robotham, M.R., Model, Z., Silver, R., Weaver, D.R. Antibodies for assessing circadian clock proteins in the rodent suprachiasmatic nucleus. PLoS One 7, e35938 (2012).

Rabbit anti-Pigment Dispersing Factor (PDF), Gift from M. Nitabach:
Control fly brains stained with this antibody displayed the expected temporal and spatial expression of the PDF neuropeptide in line with previous literature. Also, this antibody was previously validated/used in:
Nitabach, M.N, Wu, Y., Sheeba, v., Lemon, W.C, Strumbos, J., Zelensky, P.K., White, B.H, Holmes, T.C. Electrical hyperexcitation of lateral ventral pacemaker neurons desynchronizes downstream circadian oscillators in the fly circadian circuit and induces multiple behavioral periods. J Neurosci 26,479-89 (2006).

Guinea Pig anti-dPERIOD (GP73), Gift from I. Edery:
Fly clock cells stained with this antibody displayed the expected temporal and spatial expression of the dPER protein in line with previous literature. Also, this antibody was previously validated/used in:
Sidote, D., Majercak, J., Parikh, V. & Edery, I. Differential effects of light and heat on the Drosophila circadian clock proteins PER and TIM. Mol. Cell. Biol. 18, 2004–2013 (1998).
Kim, E.Y., Ko, H.W., Yu, W., Hardin, P.E., and Edery, I. A DOUBLETIME Kinase Binding Domain on the Drosophila PERIOD Protein Is Essential for Its Hyperphosphorylation, Transcriptional Repression, and Circadian Clock Function. Mol. Cell. Biol. 27, 5014–5028 (2007).

# Eukaryotic cell lines

Policy information about cell lines

| | |
|---|---|
| Cell line source(s) | WT HEK293T cells and UBR4 knockout HEK293T cells were a gift from Shashank Tripathi (Sumit K. Chanda laboratory), and were used in --> Tripathi, S. et al. (2015), Meta- and Orthogonal Integration of Influenza "OMICs" Data Defines a Role for UBR4 in Virus Budding. Cell Host Microbe 18(6): 723–735.<br><br>HEK293T cells, American Type Culture Collection, ATCC CRL-3216: https://www.atcc.org/products/crl-3216<br><br>Neuro-2a cells, American Type Culture Collection, ATCC CCL-131: https://www.atcc.org/products/all/CCL-131.aspx |
| Authentication | The CRISPR knockout cell line was authenticated by our lab by Western blot to confirm the absence of the targeted gene product. WT HEK293T and Neuro-2a cells were authenticated during cell culture on the basis of their cellular morphology. |
| Mycoplasma contamination | No signs of mycoplasma contamination were observed during microscopic examination of the cells. |
| Commonly misidentified lines (See ICLAC register) | None. |

# Animals and other organisms

Policy information about studies involving animals; ARRIVE guidelines recommended for reporting animal research

**Laboratory animals**

MICE:

The following mouse strains were purchased from The Jackson Laboratory and bred in-house to generate the appropriate genotypes for this study: homozygous ubr4fl/fl mice (JAX: 024844), homozygous Vgat-IRES-cre (Vgat-cre/cre) knockin mice (JAX: 016962); and C57BL/6J mice. Vgatcre/cre mice were bred to ubr4fl/fl mice, and a breeding colony was maintained by mating ubr4fl/fl and Vgat-cre/+;ubr4fl/+ animals. Littermate controls were used wherever possible for experiments. Vgat-cre/cre mice were also bred to C57BL/6J mice to generate Vgat-cre/+ animals. Unless otherwise specified, mice were bred and maintained on a fixed 12-h light:12-h dark (12:12 LD) schedule in which lights on and lights off corresponded to 7 am and 7 pm Eastern Standard Time, respectively. The animals were maintained at 40-60% humidity and 20-24 degrees Celsius. Lighting conditions in animal housing rooms were 100-150 lux (white light) measured at room floor level. Lighting conditions in behavioral cabinets were as defined in each experiment. Air exchanges in the animal housing rooms and behavioral cabinets were maintained at 15-20 ACH.

For behavioral experiments, male mice were set up in running-wheel cages at 5 to 8 weeks of age (if non-surgerized), or 2 weeks after stereotaxic injections,which were performed at 8 to 12 weeks of age. Five- to 8-week-old mice of both sexes were used for all tissue harvests, with the exception of those related to the stereotaxic injection experiments. In this instance, tissues were harvested from male mice at 16-20 weeks of age, at the end of the ChrA6/2 schedule.

FLIES:

All fly strains were reared on food containing agar, glucose, sucrose, yeast, cornmeal, wheat germ, soya flour, molasses, propionic acid, and Tegosept on a 12:12 LD schedule at 25 °C, 50% relative humidity. UAS and GAL4 controls were used as heterozygotes after crossing to w1118 as the wildtype strain. Unless otherwise stated, male flies were used for all experiments. Two - to -4d old female flies were used for qRT-PCR experiments, ~17-18d-old male and female flies were used for RNAscope experiments, and ~17-18d-old male flies were used for immunostaining and CD2-HRP detection experiments. The genotypes of experimental flies and controls are as follows:

w1118;UAS-poeRNAi/+;+/+ (+>poeRNAi),

UAS-Dcr2; tim-GAL4/+;+/+ (tim>Dcr2),
UAS-Dcr2; tim-GAL4/UAS-poeRNAi;+/+ (tim>Dcr2; poeRNAi),
w1118; Pdf-GAL4/+; UAS-Dcr2/+ (Pdf>Dcr2),
w1118; Pdf-GAL4/UAS-poeRNAi; UAS-Dcr2/+ (Pdf>Dcr2; poeRNAi),
elav-GAL4; UAS-Dcr2/+; +/+ (elav >Dcr2),
elav-GAL4; UAS-Dcr2/UAS-poeRNAi;+/+ (elav> Dcr2; poeRNAi),
w1118; Pdf-GAL4/UAS-CD2-HRP; UAS-Dcr2/+ (Pdf>Dcr2; CD2-HRP),
w1118; Pdf-GAL4/UAS-poeRNAi, UAS-CD2-HRP; UAS-Dcr2/+ (Pdf>Dcr2; poeRNAi, CD2-HRP).

Fly Strains:

w1118 from Bloomington RRID: BDSC_5905
UAS-Dicer2; tim-GAL4 Gift from O. Shafer N/A
Pdf-GAL4 Gift from P. Taghert N/A
elav [C155]; UAS-Dicer2 from Bloomington RRID: BDSC_25750
UAS-Dicer2 from Bloomington RRID: BDSC_24651
UAS-poe RNAi; P{KK101471}VIE-260B Vienna Drosophila Resource Center VDRC: 108296; Flybase ID:FBgn0011230
UAS-CD2-HRP from C.-H. Lee

| | |
|---|---|
| Wild animals | The study did not involve wild animals. |
| Field-collected samples | The study did not involve samples collected from the field. |
| Ethics oversight | All animal handling and experimental procedures were performed at the University of Toronto Mississauga (UTM) Animal Facility and were approved by the University of Toronto (U of T) Local Animal Care Committee, complying with guidelines established by the U of T University Animal Care Committee and the Canadian Council on Animal Care.<br><br>Drosophila melanogaster is an invertebrate organism and therefore does not require ethics approval for experimentation. |

Note that full information on the approval of the study protocol must also be provided in the manuscript.
